# Fatty Acid Composition in Blubber, Liver, and Muscle of Marine Mammals in the Southern Baltic Sea

**DOI:** 10.3390/ani10091509

**Published:** 2020-08-26

**Authors:** Dirk Dannenberger, Ramona Möller, Linda Westphal, Timo Moritz, Michael Dähne, Bianka Grunow

**Affiliations:** 1 Leibniz Institute for Farm Animal Biology, Institute of Muscle Biology and Growth, 18196 Dummerstorf, Germany; dannenberger@fbn-dummerstorf.de; 2 Albrecht Daniel Thaer-Institute for Agricultural and Horticultural Sciences, Faculty of Life Sciences, Humboldt-University Berlin, 10099 Berlin, Germany; Ramona.Moeller@agrar.hu-berlin.de; 3 Deutsches Meeresmuseum, Katharinenberg 14-20, 18439 Stralsund, Germany; Linda.Westphal@meeresmuseum.de (L.W.); Timo.Moritz@meeresmuseum.de (T.M.); Michael.Daehne@meeresmuseum.de (M.D.); 4 Institute of Zoology and Evolutionary Biology, Friedrich-Schiller-University Jena, Erbertstr. 1, 07743 Jena, Germany

**Keywords:** Baltic Sea, blubber, fatty acids, grey seals, harbour porpoise, liver, muscle

## Abstract

**Simple Summary:**

Marine mammals play an important role in marine ecosystems. However, as they are less accessible for research, relatively little is known about their physiology compared to terrestrial mammals. The stranding scheme of the Deutsches Meeresmuseum (Stralsund, Germany) continuously collects strandings and by-catches of marine mammals in the Baltic Sea in Mecklenburg-Western Pomerania. In this project, the fatty acid composition of the liver, skeletal muscles, and blubber of harbour porpoises and grey seals from the southern Baltic Sea was investigated for the first time. In the liver and blubber tissue, the values and concentrations measured for both species are consistent with studies on other marine mammals. In a direct comparison of the focus species, the skeletal muscles of harbour porpoises exhibit higher concentrations of fatty acids than those of grey seals. In the future, these studies will be extended to the entire Baltic Sea, as we suspect that fatty acid composition can be used to determine the nutritional status of the animals and thus will allow for an objective assessment of the body condition.

**Abstract:**

To date, only limited results on the fatty composition in different tissues of the top predators in the Baltic Sea are available. In the current study, tissue samples of blubber, skeletal muscle, and liver from 8 harbour porpoise (*Phocoena phocoena*) and 17 grey seals (*Halichoerus grypus)* in the Baltic Sea off Mecklenburg-Western Pomerania were included in the investigation. While the total fatty acid content in liver and blubber tissue revealed no differences between both species, the total fatty acid content of muscle tissue was significantly differentand showed higher concentrations in harbour porpoise muscle compared with grey seals. The most abundant fatty acids in the blubber of grey seals and harbour porpoises (18:1*cis*-9, 16:1*cis*-9, 16:0 and 22:6*n*-3) were present in similar quantities and ratios to each other as known from other marine top predators. If future studies can show that differences in tissue fatty acid content are caused by variation in the nutritional status, and this may lead to the development of a more objective assessment of body condition in seals and porpoises recovered via stranding schemes.

## 1. Introduction

The ecosystem and food web of the Baltic Sea is comparatively young. From a geological perspective, the Baltic Sea is a marginal sea of the Atlantic Ocean, with present time environmental conditions reaching back to only about ~7000 years ago. Grey seals (*Halichoerus grypus*) and harbour porpoise (*Phocoena phocoena*) currently are the two main mammalian predators in the wider Baltic Sea area, including the Kattegat. These two species have surpassed ringed seals (*Pusa hispida*), which were formerly the most abundant mammalian species in the Baltic Sea [1,2], harbour seals (*Phoca vitulina*), and the rarely appearing vagrant cetacean species entering from the North Sea.

Grey seal and harbour porpoise have been heavily affected by human impacts over the last centuries [3,4]. Regarded as a pest for fisheries, grey seals have been systematically hunted at the beginning of the 20th century, resulting in local eradication, e.g., in 1920, the last seal was shot in the German waters of the Baltic Sea [5,6]. Around 1900, the population of Baltic grey seals was approximately between 80,000 and 100,000 seals, which was reduced by hunting to 20,000–40,000 animals in 1940 [7,8]. The pollution of the Baltic Sea caused additional infertility of grey seals, which led to further population decline [9]. In the 1980s, about 2500 grey seals remained in the northern Baltic Sea [10]. Due to effective nature conservation management, including a Baltic wide hunting ban and the improvement of the environmental status, the number of grey seals increased steadily. Nowadays, the population has reached more than 30,000 Baltic grey seals again [11]. The total number of harbour porpoise, with a size of usually around 1.5 m, is heavily affected by bycatch in fisheries activities [12,13]. Today, the situation of these two top predators is much different: While the grey seal population increases with the recolonization of former habitats [4,11], harbour porpoises are under constant threat due to continuing bycatch impacts, decreased prey availability (e.g., [14]), and noise impacts [15,16,17]. Still, seals and porpoises are the top predators in the Baltic and therefore likely of major importance as regulatory elements for the ecosystem. Nevertheless, the exact function of these predators in the food web remains unclear.

Lipids are essential for all organisms, as they play a major role for metabolism and cell structure, such as membrane structure, cellular signal transmission, or energy storage. Marine mammals possess a specialized layer of superficial blubber providing further insulation, streamlining, buoyancy, and energy storage at the same time. The blubber layer is the most conspicuous layer of lipid deposits and was intensively investigated due to its external position and therefore simplicity of sampling also in vivo with minimal animal welfare impact. Within this thick blubber layer, numerous studies have already been carried out regarding morphology, lipid distribution, and lipid composition as well as the function of lipids for thermoregulation, energy storage, buoyancy, or streamlining [18,19,20,21,22]. In grey seals of the Baltic Sea, blubber thickness differs between age, sex, year, month, region, and health status [23]. Harbour porpoises occurring in cold to temperate waters and spotted dolphins show large differences in blubber thickness and heat conductivity [20], suggesting that small cetaceans’ blubber thickness and insulation depend highly on water temperature. The lipid composition of blubber layers therefore seems to depend on the food supply and external factors [24]. Marine mammal blubber is not homogenous and is functionally a complex tissue: The inner and outer blubber layers are significantly different, suggesting a stratification of the fatty acid composition of the layers [25]. The pattern of stratification in the marine mammals is prevalent, with increasing proportions of monounsaturated fatty acids (MUFAs) to the outer layer and increasing proportions of saturated and polyunsaturated fatty acids (SFAs, PUFAs) to the inner layer [26,27]. This variation in the fatty acid composition between the inner and outer blubber layer seems to be an indicator of different metabolic pathways, as recently shown in grey seals [28]. Due to the difficult sampling, much less is known about the lipid composition in the liver and skeletal muscles of whales and seals. Koopman described that most marine species synthesize and deposit large quantities of wax esters as storage lipids in muscle, liver, and adipose tissues [28].

Fats are of major importance to all domains of life, as they are essential parts of cellular membranes separating the cell from a potentially hostile environment [29]. Terrestrial and marine mammals do not have the ability to synthesize all of the necessary fatty acids, especially the polyunsaturated fatty acids n-3 PUFA and n-6 PUFA, in a *de novo* process that all living bacteria use to build their cell membranes. Those fatty acids have to be provided by feeding on prey items that contain high fractions of these fatty acids, which is true for fish and krill in general. For sharks, rays, and chimaeras, it has furthermore been shown that their fatty acid profiles can be used as tracers not only for their trophic guild but also for phylogeny, water temperature, and habitats [30]. Hence, taxonomically inclusive fatty acid data, currently only available to a low degree for porpoises and seals, have to be collected from different areas, such as presented here, before a meta data analysis can be conducted to conclude, for instance, on food webs [30]. In the present study, we compared the fatty acid composition of blubber, liver, and muscle tissue of the two top predators of the Baltic Sea, grey seal and harbour porpoise, to provide a background on the differences found between these species and to establish a baseline for fatty acid composition. Both species are opportunistic feeders that have an overlapping food spectrum, with cod and herring being preferred by adult porpoises and gobies playing a major role for juveniles [31], while Baltic grey seals feed on herring, sprat, cod, and sandeels [32]. In addition, they may have different prey size preferences [31,33].

## 2. Materials and Methods

### 2.1. Sample Collection

The stranding scheme of the Deutsches Meeresmuseum (Stralsund, Germany) continuously surveys strandings and bycatches of marine mammals in the Baltic Sea in Mecklenburg-Western Pomerania. This provided access to tissue samples of blubber, skeletal muscle, and liver samples, which were taken from 8 harbour porpoise and 17 grey seals (Table 1). There are no ethical conflicts in this study. Animals used in this study showed low decomposition (state 1–3) and had a good nutritional status (classes: good, moderate, bad) following approved methods taking a thin blubber layer or muscle atrophy, a hollow appearance behind the skull or dorsal fin, or clear protrusion of the lateral process of lumbar vertebrae into account [34]. Animals were necropsied or frozen immediately after delivery to the Deutsches Meeresmuseum. All animals underwent a regular necropsy protocol, thus all accompanying data, e.g., sex, size, weight, health condition, anomalies, are available via the stranding scheme database. Total length and total weight as well as the weight of the heart and liver of grey seals and harbour porpoise were measured (Table 1). In the case of the blubber, the full cross-section was sampled from the skin to the muscle to ensure full sampling of the blubber layer in the dorsal area of the last thoracic vertebra. The blubber was isolated before conduction of the lipid extraction and fatty acid ester preparation. The skeletal muscle samples were taken from the longissimus dorsi and psoas muscle to ensure standardization of the sampling process and also to ensure that for pinnipeds and cetaceans the same muscles with similar functionality (aquatic locomotion) were used following [35]. Directly after, sampling tissues were stored again at −20 °C until further analysis. The grey seals used in this study were fully foraging on fish and independent of their mother. Grey seals in the Baltic Sea have been reported to be born in February to early March [36]. All seals with <1.5 m body length were found after May, ensuring that the weaning period of up to four weeks and the post weaning fast of approximately 4 weeks had already ended. A good nutritional status of these animals also indicated that the post weaning fast was finished. Three porpoises in the study were below 1 m in length and were therefore regarded as neonates [37] depending on the lactating mother.

### 2.2. Lipid Extraction and Fatty Acid Ester Preparation (Blubber, Muscle, Liver)

After homogenization of slightly thawed samples and the addition of nonadecanoic acid (C19:0) as an internal standard, total lipids were extracted in duplicate using chloroform/methanol (2:1, *v/v*) and the Ultra Turrax T25 (IKA, Staufen, Germany) at 3 × 15 s, 15,777 g, and room temperature. For lipid extraction, approximately 2 g of muscle, 2 g of liver, and 1 g of blubber were used. The detailed sample preparation procedure of animal tissues was previously described [38]. Briefly, the final extraction mixtures were stored at 5 °C for 18 h in the dark and subsequently washed with 0.02% CaCl_2_ solution. All of the solvents contained 0.005% (weight/volume (*w/v*)) of t-butylhydroxytoluene to prevent the oxidation of PUFAs. The organic phase was separated and dried with a mixture of Na_2_SO_4_ and K_2_CO_3_ (10:1, weight/weight (*w/w*)), and the solvent was subsequently removed under gentle nitrogen at room temperature. The lipid extracts were redissolved in 300 μL of toluene, and a 25-mg aliquot was used for methyl ester preparation. Total lipids were stored at −18 °C until transmethylation of fatty acids. For transmethylation, 2 mL of 0.5 M sodium methoxide in methanol were added to the lipid extracts, which were shaken in a 60 °C water bath for 10 min. Subsequently, 1 mL of 14% boron trifluoride in methanol was added to the mixture, which was then shaken for an additional 10 min at 60 °C. Saturated NaHCO_3_ solution (2 mL) was added, and the fatty acid methyl esters (FAMEs) were extracted twice with 2 mL of *n*-hexane. The *n*-hexane extracts were dried with a mixture of Na_2_SO_4_ and K_2_CO_3_ (10:1, *w/w*). After filtration, the extracts were reduced to dryness using a vacuum centrifuge (438 g, 30 °C, 30 min). The FAMEs were resuspended in 100 µL of *n*-hexane and stored at −18 °C until use for high-resolution gas chromatography (HR-GC) analysis.

### 2.3. Fatty Acid Analysis 

The fatty acid analysis of the lipids was performed using capillary HR-GC with a CP-Sil 88 CB column (100 m × 0.25 mm, Agilent, Santa Clara, CA, United States) that was installed in a PerkinElmer gas chromatograph CLARUS 680 with a flame ionization detector and split injection (PerkinElmer Instruments, Waltham, Massachusetts, U.S.A.). The detailed GC conditions were recently described [39]. Briefly, the initial oven temperature was 150 °C, which was held for 5 min. Subsequently, the temperature was increased to 175 °C followed by 200 °C at a rate of 2 °C min^−1^ and held for 10 min. Finally, the temperature was increased to 225 °C at a rate of 1.5 °C min^−1^ and held for 25 min. Hydrogen was used as the carrier gas at a flow rate of 1 mL min^−1^. The split ratio was 1:20, and the injector and detector were set at 260 and 280 °C, respectively. The quantification of fatty acids was performed using C19:0 as the internal standard [39]. For the calibration procedure, the reference standard mixture “Sigma FAME” (Sigma-Aldrich, Deisenhofen, Germany), the methyl ester of C18:1*cis*-11, C22:5*n*-3, and C18:2*cis*-9,*trans*-11 (Matreya, State College, PA, USA), C22:4*n*-6 (Sigma-Aldrich, Deisenhofen, Germany), and C18:4*n*-3 (Larodan, Limhamn, Sweden) were used. The five-point calibration of single fatty acids ranged between 16 and 415 µg/mL and was assessed after GC analysis of five samples. Fatty acid concentrations are displayed as mg/100 g tissue.

### 2.4. Data Analysis

Pearson correlation coefficients between the morphometric data total body weight, body length, heart weight, and liver weight were calculated to validate normal growth of the animals. By using the Mann–Whitney analysis, it was also ensured that the fatty acid composition of the animals examined in this study was not influenced by age, time of death, and degree of decay (data not shown). For statistical analysis of the fatty acid concentrations, the software SAS 9.4 (SAS Institute Inc., Cary, NC, USA) was used. Graphs were performed using the software GraphPad Prism 8. All data are expressed as mean ± SEM. In a first step, a Pearson correlation analysis with the values body length and body weight, thickness of the blubber layer, and total fatty acid content was performed. A significant level of *p* < 0.05 was defined for this correction analysis. In a second step, a Mann–Whitney U-Test was used to compare the two species with regard to their differences in the fatty acid composition of liver, muscle, and blubber tissue. In total, we performed 20 comparisons per tissue. Therefore, we corrected the significance level for multiple testing. A *p*-value of <0.0025 was defined as significant for this analysis. Since the distribution of the sexes within the species was not uniform, the sexes were not taken into account in the analysis. Only the fatty acids presented in concentrations >0.05 mg/100 g tissue were included in the statistical analyses.

## 3. Results

### 3.1. Morphometric Data

Morphometric examinations of the investigated animals showed that their organ weights depend on the animals’ size. Thus, in both species, almost linear relationships between the factors total body weight, body length, heart weight, and liver weight were shown (Table 2 and Figure S1). Large seals, however, showed a slightly stronger increase in body weight than in body length (Figure S1a). A similar trend was not obvious for heart and liver weight, which rather follow a continuous correlation with body weight (Figure S1b,c). Pearson correlation analysis, with the values body length and body weight, thickness of the blubber layer, and total fatty acid content (mg/g) of the tissues, showed no correlations between these values (total length/fatty acid content: grey seals: r = 0.06; *p* = 0.85 and harbour porpoise: r = 0.64; *p* = 0.09; fatty acid content/ thickness off at layer: harbour porpoise r = 0.35; *p* = 0.40). We can therefore assume that neither the age of the animals (represented by the body length) nor the time of death in the course of the year had any influence on the analysis. Therefore, this was not considered any further in the present study. Based on this and due to the low number of 17 grey seals and 8 harbour porpoises in total, no differentiation between sex and age was performed in the statistical analyses.

### 3.2. Fatty Acid Concentrations in Tissues of Grey Seals and Harbour Porpoise

**Figure 1:**
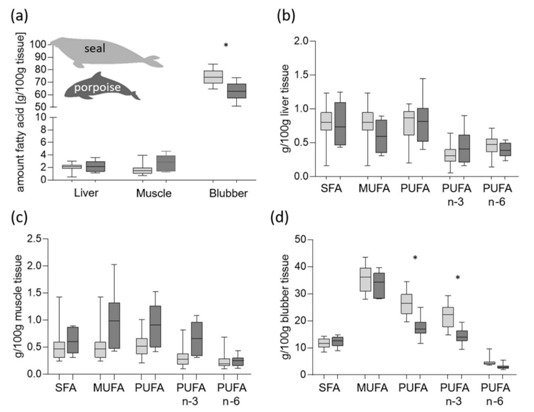
Total fatty acid concentration in the investigated tissues (**a**) and group fatty acid concentrations (sum) in liver (**b**), skeletal muscle (**c**), and blubber (**d**) of grey seals (light grey boxes) and harbour porpoise (dark grey boxes), * significant different between both species (*p* < 0.0025).

While the total fatty acid content in liver and muscle tissue revealed no differences between both species, the total fatty acid content of blubber tissue was significantly different, and showed higher concentrations in grey seals compared to harbour porpoises: 74.4 ± 1.5 g/100 g vs. 63.1 ± 2.6 g/100 g fresh tissue, *p* = 0.0018 (Figure 1a, Table S1). The sums of the fatty acid concentration (sum SFA, sum MUFA, sum PUFA, sum n*-*3 PUFA, sum n-6 PUFA) in the liver and muscle of both species showed no significant differences (Figure 1b,c, Table S1). In contrast, the sums of the fatty acid concentration in blubber tissue were significantly different between both species. Higher contents of sum PUFA and sum n-3 PUFA were detected in grey seals (e.g., sum PUFA: 26.78 ± 1.2 g/100 g fresh blubber tissue vs. 17.5 ± 1.4 g/100 g fresh blubber tissue; Figure 1d, Table S1). The sum MUFA and sum SFA concentrations of all three investigated tissues (muscle, liver, and blubber) of grey seals and harbour porpoises were not significantly different. In both species, the MUFA concentrations had the highest values in blubber tissue compared to the other sum fatty acid groups (Figure 1d).

**Figure 2:**
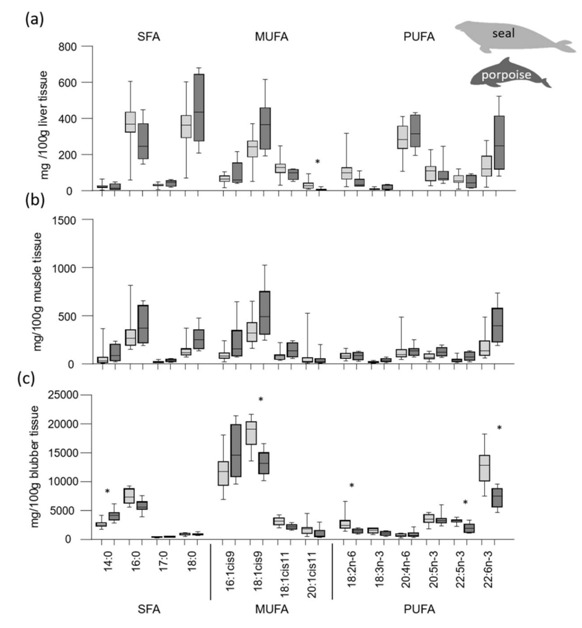
Single fatty acid concentrations (mg/100 g fresh tissue) in liver (**a**), skeletal muscle (**b**), and blubber tissue (**c**) of grey seals (light grey boxes) and harbour porpoise (dark grey boxes), * significant different between both species (* *p* < 0.0025).

The results of selected single fatty acid concentrations (mg/100 g fresh tissue) of both species are presented in Figure 2. No significant differences were detected in the muscle and liver of grey seals and harbour porpoise, except higher 20:1*cis*11 concentrations in grey seal compared to harbour porpoise: 29.8 ± 5.6 mg/100 g vs. 8.1 ± 2.3 mg/100 g fresh liver tissue (20:1*cis*11), respectively (Figure 2a, Tables S1 and S2). The single fatty acid concentration’s significance in blubber tissue confirms the sum fatty acid concentrations’ results in that significant differences between the species could be shown for a number of single fatty acids (Figure 2b,c, Tables S1 and S2). In blubber tissue, a high number of fatty acids were significantly different between grey seals and harbour porpoise, e.g., 18:1*cis*-9 (18.4 ± 0.6 g/100 g vs. 13.1 ± 0.8 g/100 g fresh tissue; *p* < 0.0006) and 22:6*n*-3 (12.8 ± 0.8 g/100 g vs. 7.2 ± 0.6 g/100 g fresh tissue; *p* < 0.0002) (Figure 2c, Tables S1 and S2). In contrast, single PUFA concentrations (18:3*n*-3, 20:5*n*-3, 22:5n-3) in the muscle and liver of grey seal and harbour porpoise were not significantly different.

## 4. Discussion

To date, only limited results on fatty composition in tissues of the top predators in the Baltic Sea are available, with the exception of blubber in grey seals [27,40]. As we present here a preliminary study on the fatty acid composition in different tissues of grey seals and harbour porpoises in the southern Baltic Sea, the study has a descriptive character based on the limited number of animals in both species. Based on the low number of 17 grey seals and 8 harbour porpoises in total and no differentiation between sex and age, the statistical significance level for the analysis of fatty acid analysis in the tissues was reduced to *p* < 0.0025. The most abundant fatty acids in the blubber of grey seals and harbour porpoises 18:1*cis*-9, 16:1*cis*-9, 16:0, and 22:6*n*-3 could be identified and quantified in a similar pattern to the distribution in other marine top predators [26]. The fatty acids 18:1*cis*-9, 16:1*cis*-9, 16:0, and 22:6*n*-3 were also reported as the major fatty acids in the blubber of seals (grey, harbour, harp, Australian fur, crabeater) in other studies from sea areas, e.g., of eastern Canada, south-east Australia, or the Antarctic Peninsula [26,41,42]. These fatty acids, except 22:6*n*-3, seem to be predominantly endogenous biosynthesized and thus not influenced by the type of prey of the predator [43]. In addition, 18:1*cis*-9, 16:0, and 16:1*cis*-9 are not only ubiquitous in marine mammals but also the most abundant main fatty acids in the tissues of terrestrial mammals like pigs and cattle [44,45]. Besides these, the PUFA 22:6*n*-3, 20:5*n*-3, and 22:5*n*-3 were also measured in higher concentrations in the blubber of grey seals and harbour porpoises compared with other fatty acids. Grey seals and harbour porpoises share similar food items, habitat and geographic distributions. Porpoises and grey seals feed on a variety of food items. While porpoises prefer smaller food items, such as juvenile cod (*Gadus morhua*), herring (*Clupea haraengus*), and gobiids [31], grey seals in the northern parts of the Baltic Sea feed mainly on herring, sprat (*Sprattus sprattus*), and common whitefish (*Coregonus maraena* often named *C. lavaretus* in various publications) [43]. This may explain some of the variation found, especially in the fatty acid composition of the blubber layer.

PUFAs originate from the diet and occur mainly in krill and other crustaceans, and were similarly detected in higher proportions in Arctic seals, like ringed seals [41,46]. For porpoises and seals, that may indicate a second-order intake of crustaceans via bottom-feeding fish (e.g., cod) that in turn feed on crustaceans or pelagic fish that feed on krill. In general, blubber tissue in marine mammals has two important biological functions: It firstly acts as an energy storage and secondly as a thermal regulator [47]. By the use of stratification analysis, it could be revealed that the fatty acid composition in the blubber of seals (grey seals, crabeater seals) shows large differences in biochemistry between the outer and inner blubber layer [26,27]. The inner blubber layer, containing high lipid proportions of longer chain SFAs and PUFAs, seems to reflect the fatty acid composition of the diet of the seals more than the outer blubber layer. In contrast, in the outer layer, the MUFAs are more enriched, which do not seem to be readily mobilized by the seals and therefore accumulated in the blubber tissue [26]. These higher MUFA concentrations suggest a more functional role of thermoregulation of the outer blubber layer of seals [27,48]. In the current study investigating grey seals and harbour porpoises of the southern Baltic Sea, no investigations were performed on the different fatty acid composition in the inner and outer blubber layer. Compared to the grey seals, there is only limited information on the fatty acid composition and topography in the blubber of harbour porpoise in the northeast Atlantic available (summarized in [49]). The results of the current study indicated significant differences in the fatty acid concentrations in the blubber of grey seal and harbour porpoise for MUFAs and PUFAs as well as for SFAs. For example, the concentrations of 16:0, 18:1*cis*-9, 18:2*n*-6, 18:3*n*-3, and 22:5*n*-3 in the blubber of harbour porpoises were lower compared with the contents in grey seals. Based on the idea that the lipid composition in blubber mainly depends on the food supply and external factors, on the one hand, variations in the fatty acid concentrations in blubber between grey seal and harbour porpoise may be explained by differences in diet composition [24]. On the other hand, the endogenous *de novo* biosynthesis of fatty acids in the blubber of marine mammals seems to play only a subordinate role, except for the MUFAs 16:1*cis*-9 and 18:1*cis*-9 in the inner layer. In general, the proportions of total SFA and PUFA in the blubber of harbour porpoises in the Baltic Sea from the current study (Tables S1 and S2) are comparable with those in the North Sea [50]. In contrast to the blubber, the fatty acid composition in the liver and muscle of grey seals and harbour porpoises showed no significant differences, except for 20:1*cis*-11. The most abundant fatty acids in the liver of grey seal and harbour porpoise were 16:0, 18:0, 20:4*n*-6, 18:1*cis*-9 and 18:0, 18:1*cis*-9, 20:4*n*-6, 16:0, respectively. The fatty acids with the highest concentrations in the muscle of grey seal and harbour porpoise were 18:1*cis*-9, 16:0, 22:6*n*-3, 18:0 and 18:1*cis*-9, 22:6*n*-3, 16:0, 18:0, respectively. The fatty acids in the lipids of muscle of predominantly PUFAs of both species seem to be underlying metabolic modifications before deposition in the tissues [27,31]. It has been shown that the MUFA and PUFA levels in the skeletal muscle of diving mammals (e.g., Weddell seals) change during growth, potentially involved in energy production under extreme pressure [51,52]. Besides the effects of PUFAs in inflammatory and immune processes in the development of diving mammals, fatty acids also play a role in energy metabolism during adaptation to diving activities. PUFA levels are elevated in the skeletal muscle membrane of the smaller pup and decrease in the juvenile towards the adult animals, as shown for Weddell seals [51,52]. In contrast, the levels of SFA and MUFA increase in the skeletal muscle of adult animals as a possible protection against reactive oxygen species damage due to high rates of sustained lipid metabolism associated with the end of dives [51,52]. To our knowledge, only a limited number of studies on fatty acid *de novo* lipogeneses and lipid metabolism in different tissues of marine mammals have been conducted to date [27]. Marine mammals typically consume a high-fat low-carbohydrate diet and seem to show lower rates of fatty acid *de novo* synthesis compared to terrestrial mammals [53]. The primary sites of *de novo* fatty acid synthesis in rodents and humans is the liver; however, in many other species (e.g., cattle, pigs, and dogs), the adipose tissues and mammary gland (e.g., in cattle and sheep) are the main sites of lipogenesis [54]. It is generally known that in cattle and pig, muscle and adipose tissues is one of the key factors regulating the *de novo* synthesis of fatty acids and deposition is the expression of lipogenic enzymes. Dietary n-3 PUFA intervention led to a suppression of key-lipogenic transcription factors (SREBP-1c) and enzymes involved in fatty acid *de novo* synthesis (ACC, FAS) monodesaturation (SCD-1) and polyunsaturation (d5-desaturase, d6-desaturase) in pigs and cattle [55,56].

One particular shortcoming is our approach to age estimation based on body length, which could be improved using teeth aging methods [57,58,59] in the future. In addition to these obstructions, the use of stranded and bycaught animals may introduce a bias, since a cause of death could not be diagnosed for all of the animals. However, for porpoises, this deficit cannot be easily overcome, since samples from life animals (biopsies) cannot be acquired in most places. Especially, for the Baltic Sea, that information may be crucial, since the population is at the brink of extinction [13]. For grey seals, however, in the future, our results may be compared to biopsies of live animals.

With the current study, we could show that between grey seals and harbour porpoises, a different fatty acid composition of the tissue occurred predominantly in blubber tissue, although not in muscle and liver tissue. One key factor in monitoring marine mammal populations is the regular checking of their health status. If future studies can show that these alterations are caused by a change in the fatty acid proportion and composition, this may lead to the development of a more objective assessment of nutritional status in seals and porpoises. In addition, detailed lipidomic/metabolomic studies of grey seal/harbour porpoise tissues according to sex and age, including the food supply, would be a value complementation to the assessment of their nutritional status.

## Figures and Tables

**Table 1:** Body length and weights of the body, liver, and heart of harbour porpoise and grey seals used in the present study, ID—identification number of stranding scheme.

ID	Species	Sex	Body Length (m)	Body Weight (kg)	Liver (kg)	Heart (kg)
B64/17	Grey seal	male	2.24	139.8	4.27	0.96
B63/17	Grey seal	male	2.1	103.0	4.47	0.80
B56/17	Grey seal	male	2.03	90.0	2.99	0.63
M20/18	Grey seal	male	*	*	1.80	0.44
B61/17	Grey seal	male	2.03	75.5	4.65	0.82
B65/17	Grey seal	male	2.13	156.0	4.27	0.90
B68/17	Grey seal	male	2.33	148.0	5.03	0.82
B38/16	Grey seal	male	1.32	31.4	1.50	0.30
B78/17	Grey seal	male	2.16	116.8	2.91	0.62
B07/14	Grey seal	female	1.8	71.6	2.54	0.54
B75/17	Grey seal	male	2.11	106.4	3.00	0.63
B08/17	Grey seal	male	1.21	29.0	0.68	0.18
B58/16	Grey seal	male	2.07	101.8	3.80	0.82
B74/17	Grey seal	male	2.34	190.8	5.35	1.00
B50/15	Grey seal	male	2.04	145.2	4.81	0.75
M157/18	Grey seal	male	2.26	153.6	5.34	0.82
M47/18	Grey seal	male	2.07	94.8	2.60	0.62
Mean ± S.E.M.	Grey seals		2.02 ± 0.08	109.6 ± 11.24	3.53 ± 0.34	0.69 ± 0.05
B32/15	Harbour porpoise	female	0.99	14.8	0.45	0.07
B04/17	Harbour porpoise	male	1.07	24.2	0.54	0.15
M02/18	Harbour porpoise	female	1.2	34.0	0.65	0.20
B68/16	Harbour porpoise	female	0.94	12.8	0.33	0.08
B65/13	Harbour porpoise	female	1.17	25.2	0.56	0.18
B46/17	Harbour porpoise	male	1.47	49.4	0.96	0.25
B39/13	Harbour porpoise	female	1.6	56.6	2.21	0.34
B36/16	Harbour porpoise	female	1.69	61.8	2.73	0.39
Mean ± S.E.M.	Harbour porpoises		1.31 ± 0.10	34.85 ± 6.69	1.05 ± 0.32	0.21 ± 0.04

* Total length and weight could not be measured because animals head was missing.

**Table 2:** Pearson correlation coefficient between total weight, total length, and weight of the liver as well as the heart in grey seals (n = 17) and harbour porpoise (n = 8).

Morphometric Data	Total Weight	Total Length	Weight Liver	Weight Heart
Grey seal				
total weight	1	0.88	0.84	0.85
total length	0.88	1	0.88	0.91
weight liver	0.84	0.88	1	0.94
weight heart	0.85	0.91	0.94	1
Harbour porpoise				
total weight	1	0.99	0.88	0.97
total length	0.99	1	0.90	0.96
weight liver	0.88	0.90	1	0.90
weight heart	0.97	0.96	0.90	1

## Supplementary Materials

The following are available online at https://www.mdpi.com/2076-2615/10/9/1509/s1, Figure S1: Overview of weight and body size in studied specimens. Each individual animal is represented by a colour.; circle – grey seals; triangle – harbour porpoises; Table S1: Fatty acid concentrations (mg/100g fresh tissue) in muscle, liver and blubber of grey seals and harbour porpoises, only fatty acids > 0.05 mg/100g tissue were included; Table S2: Fatty acid composition (% of total fatty acids) in muscle, liver and blubber of grey seals and harbour porpoises, only fatty acids > 0.01% tissue were included
