# Supplementary material for: Fatty Acid Composition in Blubber, Liver, and Muscle of Marine Mammals in the Southern Baltic Sea
Source: Animals (Basel). 2020 Aug 26;10(9):1509. doi: 10.3390/ani10091509 (PMC7552294; doi:10.3390/ani10091509)
Supplement: Supplementary file 1 [file animals-10-01509-s001.pdf]

animals

Article

Fatty Acid Composition in Blubber, Liver, and Muscle of Marine Mammals in the Southern Baltic Sea

Dirk Dannenberger [1], Ramona Möller [2], Linda Westphal [3], Timo Moritz [3,4], Michael Dähne [3] and Bianka Grunow [1,*]

[1] Leibniz Institute for Farm Animal Biology, Institute of Muscle Biology and Growth, 18196 Dummerstorf, Germany; dannenberger@fbn-dummerstorf.de (D.D.)
[2] Albrecht Daniel Thaer-Institute for Agricultural and Horticultural Sciences, Faculty of Life Sciences, Humboldt-University Berlin, 10099 Berlin, Germany; Ramona.Moeller@agrar.hu-berlin.de (R.M.)
[3] Deutsches Meeresmuseum, Katharinenberg 14-20, 18439 Stralsund, Germany; Linda.Westphal@meeresmuseum.de (L.W.); Timo.Moritz@meeresmuseum.de (T.M.); Michael.Daehne@meeresmuseum.de (M.D.)
[4] Institute of Zoology and Evolutionary Biology, Friedrich-Schiller-University Jena, Erbertstr. 1, 07743 Jena, Germany
* Correspondence: grunow@fbn-dummerstorf.de

Received: 17 June 2020; Accepted: 21 August 2020; Published: 26 August 2020

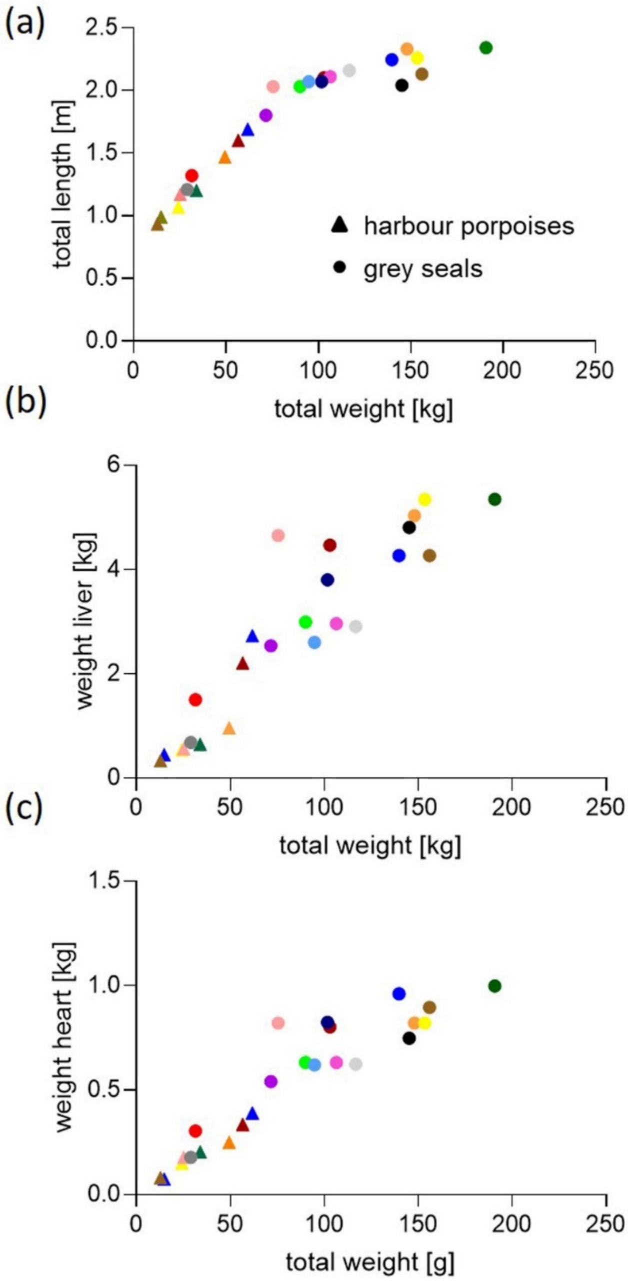

Figure S1. Overview of weight and body size in studied specimens. Each individual animal is represented by a colour.; circle – grey seals; triangle – harbour porpoises.

.

Table S1. Fatty acid concentrations (mg/100g fresh tissue) in muscle, liver and blubber of grey seals and harbour porpoises, only fatty acids > 0.05 mg/100g tissue were included.

	Muscle				Liver				Blubber				P-values		
Fatty acids (mg/100g tissue)	Grey seals (n=16)		Harbour porpoise (n=8)		Grey seals (n=15)		Harbour porpoise (n=7)		Grey seals (n=16)		Harbour porpoise (n=8)		muscle	liver	blubber
	MEAN	SEM	MEAN	SEM	MEAN	SEM	MEAN	SEM	MEAN	SEM	MEAN	SEM			
C14:0	53.3	21.9	107.6	30.0	23.6	3.7	20.3	6.4	2578.9	141.5	4121.0	356.8	0.0500	0.3413	0.0006
C16:0	307.5	44.4	396.2	65.8	354.9	34.2	267.7	41.2	7397.0	323.6	5692.5	385.1	0.1779	0.1693	0.0059
C17:0	17.1	2.7	31.2	5.0	29.3	2.5	39.7	6.0	377.4	16.6	383.4	35.9	0.0169	0.1922	0.6682
C18:0	134.0	17.7	263.4	41.3	351.6	32.4	463.0	69.0	847.6	43.3	841.2	79.9	0.0027	0.2174	0.3272
C16:1*cis*-9	91.2	15.1	228.7	69.5	63.4	5.7	94.8	25.6	11530.0	682.9	15036.8	1564.8	0.0235	0.6982	0.0275
C18:1*cis*-9	338.9	34.7	545.4	96.0	236.3	20.3	363.0	56.9	18407.4	598.7	13102.2	772.0	0.0576	0.0618	0.0006
C18:1*cis*-11	75.6	11.5	139.6	25.1	127.0	12.5	88.6	10.5	3140.1	175.8	2180.2	158.6	0.0200	0.0264	0.0048
C20:1*cis*-11	65.7	31.6	47.0	22.2	29.0	5.6	8.1	2.3	1770.8	261.2	987.5	330.6	1.0000	0.0017	0.0200
C18:2*n*-6	79.6	8.7	80.0	13.5	109.1	18.2	47.5	11.6	2689.6	308.6	1432.5	134.8	0.8065	0.0182	0.0022
C18:3*n*-3	14.6	2.2	33.9	7.2	8.4	1.1	13.7	4.9	1484.3	110.4	992.2	122.8	0.0120	0.9158	0.0200
C20:4*n*-6	133.5	27.7	134.4	20.1	284.6	21.9	313.7	34.2	651.4	61.9	838.1	207.3	0.2446	0.4592	0.6242
C20:5*n*-3	70.1	8.4	122.3	16.2	105.0	14.8	93.3	26.8	3524.5	199.3	3444.1	388.5	0.0101	0.4592	0.4260
C22:5*n*-3	34.1	6.1	74.1	15.6	58.7	8.2	53.6	12.4	3128.7	89.2	1928.7	276.2	0.0200	0.6468	0.0022
C22:6*n*-3	169.8	27.3	408.4	68.2	128.9	18.4	258.2	60.9	12769.1	797.8	7245.2	623.1	0.0027	0.0842	0.0008
[1]Sum SFA	532.3	80.7	833.2	132.7	784.4	72.2	806.8	119.2	11715.0	465.8	12336.4	681.2	0.0662	0.9719	0.4624
[2]Sum MUFA	598.9	85.1	1005.7	195.8	477.2	39.7	568.1	89.3	35926.4	1236.8	33278.2	1619.1	0.0576	0.5491	0.2207
[3]Sum PUFA	537.2	58.0	900.9	141.2	780.0	61.6	819.7	132.1	26748.2	1155.0	17489.8	1378.1	0.0321	0.9719	0.0005
[4]Sum *n*-3 PUFA	300.4	44.2	655.3	105.7	308.3	39.5	424.5	98.5	22021.3	1159.8	14215.6	1075.8	0.0048	0.3413	0.0009
[5]Sum *n*-6 PUFA	232.6	35.4	243.8	35.6	462.3	33.7	391.9	39.2	4605.6	360.2	3073.3	369.0	0.6241	0.1922	0.0027
[6]Sum fatty acids	1668.4	220.2	2739.8	452.1	2041.6	163.7	2194.6	332.3	74390.1	1502.9	63104.5	2613.2	0.0500	0.8601	0.0018

[1]SFA=C10:0+C11:0+C12:0+C13:0+C14:0+C15:0+C16:0+C17:0+C18:0+C20:0+C21:0+C22:0+C23:0+C24:0;.[2]MUFA=C14:1+C15:1+C16:1+C17:1+C18:1t+C18:1c9+C18:1c11+C22:1+C24:1; [3]PUFA=Σn-3+Σn-6FA+c9,tr11CLA+C18:2t; [4]Σn-3FA=C20:3n-3+C22:6n-3+C22:5n-3+C20:5n-3+C18:4n-3+C18:3n-3; [5]Σn-6FA=C22:2n-6+C20:2n-6+C18:3n-6+C22:4n-6+C20:3n-6 +C18:2n-6+C20:4n-6; [6]Σ fatty acids= sum SFA+sum MUFA+sum PUFA.

Table S2. Fatty acid composition (% of total fatty acids) in muscle, liver and blubber of grey seals and harbour porpoises, only fatty acids > 0.01 % tissue were included

Fatty acids (g/100g)	Muscle				Liver				Blubber				P-values		
	Grey seals (n=16)		Harbour porpoise (n=8)		Grey seals (n=15)		Harbour porpoise (n=7)		Grey seals (n=16)		Harbour porpoise (n=8)		muscle	liver	blubber
	MEAN	SEM	MEAN	SEM	MEAN	SEM	MEAN	SEM	MEAN	SEM	MEAN	SEM			
C14:0	2.4	0.5	3.5	0.6	1.1	0.1	0.9	0.2	3.5	0.2	6.5	0.5	0.0758	0.2746	0.0001
C16:0	18.4	0.4	14.6	0.4	17.0	0.7	12.4	0.7	10.0	0.5	9.0	0.5	0.0003	0.0008	0.3272
C17:0	1.0	0.0	1.2	0.1	1.4	0.0	1.8	0.1	0.5	0.0	0.6	0.0	0.0982	0.0008	0.0982
C18:0	8.5	0.6	10.2	1.0	17.1	0.5	21.1	1.2	1.1	0.1	1.3	0.1	0.1984	0.0013	0.2446
C16:1*cis*-9	5.1	0.3	7.5	1.2	3.2	0.3	4.1	0.7	15.5	0.8	23.8	2.2	0.0912	0.3067	0.0048
C18:1*cis*-9	21.1	0.6	20.0	0.8	11.6	0.3	16.6	0.9	24.8	0.7	20.9	1.1	0.1984	0.0004	0.0120
C18:1*cis*-11	4.6	0.3	5.1	0.3	6.4	0.5	4.2	0.4	4.2	0.2	3.5	0.3	0.1984	0.0034	0.0982
C20:1*cis*-11	2.9	0.8	1.6	0.7	1.5	0.2	0.4	0.1	2.5	0.4	1.6	0.5	0.0576	0.0005	0.1113
C18:2*n*-6	5.2	0.5	2.9	0.2	5.5	0.9	2.1	0.3	3.6	0.4	2.3	0.2	0.0022	0.0008	0.0071
C18:3*n*-3	0.9	0.1	1.2	0.1	0.4	0.1	0.6	0.1	2.0	0.1	1.6	0.2	0.0500	0.6982	0.0576
C20:4*n*-6	8.3	1.0	5.3	0.7	14.8	1.1	15.3	1.5	0.8	0.1	1.3	0.3	0.0321	0.8051	0.1779
C20:5*n*-3	4.3	0.3	4.7	0.3	5.1	0.5	4.1	0.6	4.7	0.2	5.4	0.4	0.3583	0.1922	0.2703
C22:5*n*-3	2.0	0.2	2.6	0.2	2.7	0.3	2.4	0.5	4.2	0.1	3.0	0.4	0.0500	0.5491	0.0235
C22:6*n*-3	10.2	0.8	15.1	0.8	5.9	0.5	10.9	1.1	17.1	0.9	11.5	1.0	0.0015	0.0013	0.0033
[1]Sum SFA	31.5	0.7	30.0	0.9	37.9	1.0	36.9	1.3	15.8	0.7	19.6	0.8	0.8065	0.6468	0.059
[2]Sum MUFA	35.4	0.7	35.8	1.6	23.6	0.8	25.9	1.2	48.3	1.4	52.8	1.6	0.9025	0.1296	0.0662
[3]Sum PUFA	33.1	0.8	33.5	1.2	38.5	0.9	37.2	0.8	35.9	1.2	27.6	1.7	0.7595	0.1922	0.0027
[4]Sum *n*-3 PUFA	18.0	1.0	24.2	0.7	14.4	1.1	18.1	1.5	29.5	1.3	22.5	1.3	0.0018	0.0842	0.0048
[5]Sum *n*-6 PUFA	14.8	1.2	9.3	0.6	23.7	1.4	18.9	1.4	6.2	0.5	4.8	0.5	0.0040	0.0526	0.0321

Footnote 1-5, see Table S1.

© 2020 by the authors. Licensee MDPI, Basel, Switzerland. This article is an open access article distributed under the terms and conditions of the Creative Commons Attribution (CC BY) license (http://creativecommons.org/licenses/by/4.0/).
